# Development of a Diagnostic Tool for Elbow Instability Combining 4D-CT Imaging and Mechanical Load Analysis

**DOI:** 10.1177/11795972261472224

**Published:** 2026-08-10

**Authors:** Jonas Gehring, Fabian Forster, Markus Meindl, Martin Maerz, Michael Simon, Nina Renner, Michael Uder, Tobias Pogarell, Lars Braeuer, Mario Perl, Lucas Mangold

**Affiliations:** 1 28452 Department of Power Electronics, Fraunhofer Institute for Integrated Systems and Device Technology IISB, Erlangen, Germany; 2 Department of Power Electronics at Friedrich-Alexander-Universität Erlangen-Nürnberg, Nürnberg, Germany; 3 Department of Orthopaedics and Trauma Surgery, University Hospital of Erlangen, 9171 Friedrich-Alexander-University Erlangen-Nuremberg, Erlangen, Germany; 4 Department of Radiology, University Hospital Erlangen, 9171 Friedrich-Alexander-Universität Erlangen-Nürnberg, Erlangen, Germany; 5 Institute of Functional and Clinical Anatomy, 9171 Friedrich-Alexander-Universität (FAU) Erlangen-Nürnberg, Erlangen, Germany

**Keywords:** elbow instability, diagnostic tool, 4D-CT imaging, mechanical load analysis, Melbo

## Abstract

**Background:**

Diagnosis of elbow instability in clinical practice commonly relies on manual stress testing by the physician with or without combination of fluoroscopy or ultrasound, which may result in variable force application and subjective interpretation. A standardized method for applying controlled mechanical loading during dynamic imaging may improve reproducibility.

**Objective:**

The objective of this study was to develop and perform initial cadaveric feasibility testing of a CT-compatible diagnostic device for device-guided assessment of elbow instability with simultaneous force and positional measurement.

**Design:**

Technical development study with initial cadaveric feasibility testing.

**Methods:**

Building upon the initial prototype the present paper, describes the development of MELBO the follow-up prototype with adjusted kinematics and improved stability, the validation of the force sensor is addressed, and the first cadaver test results are presented.

**Results:**

The MELBO diagnostic tool enabled a controlled device-guided elbow motion during 4D-CT acquisition and allowed simultaneous recording of force and positional data. The acquired dynamic CT datasets were suitable for qualitative assessment of elbow joint stability. No relevant device-related imaging artifacts were observed.

**Conclusion:**

The present study demonstrates the technical feasibility of device-guided simultaneous force and positional measurement during 4D-CT using the MELBO diagnostic tool. These findings provide the basis for further cadaveric studies and future clinical investigations aimed at quantitative assessment of elbow instability under standardized loading conditions.

## Introduction

After the shoulder, the elbow joint is the second most dislocated joint.^1^ A common cause is axial overloading of the forearm, for example, due to a fall, which can damage the stabilizing ligamentous apparatus around the elbow joint, resulting in joint instability and subsequent dislocation.^2^ Without correct diagnosis the instability can lead to arthrosis, elbow stiffness and pain.^3-5^

The current diagnostic process involves a physician's manual arm examination, combined with fluoroscopy and tomographic imaging methods. The indication for operative treatment is still discussed between medical experts.^1,6^ The treatment for low- and high-grade instabilities is somehow clear but for patients in between sometimes the only way to make a decision goes through an arthroscopic surgery. The clinical assessment of chronic elbow instability is facing the same barriers.^7^

**Figure 1.**
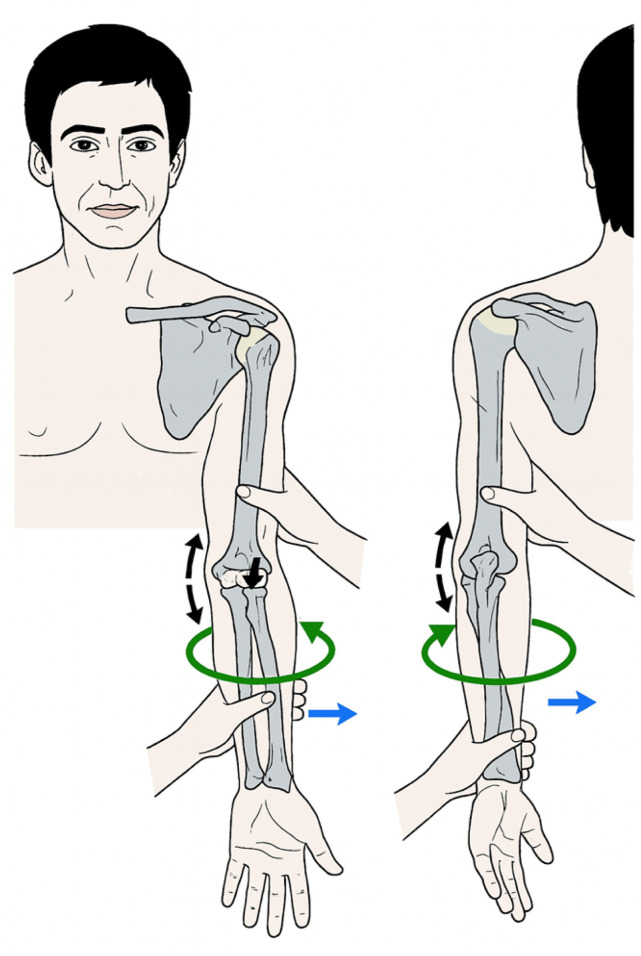
Manual execution of the valgus stress test (left) and the varus stress test (right) by a physician, adapted from.^1^ During valgus stress (black arrows, left) the forearm is fully supinated (green arrow, left) and during varus stress (black arrows, right) the forearm is pronated (green arrow, right). This load is to be applied by the developed device, and the acting forces (blue arrows) are to be measured

To assess the instability of the joint, for example the so-called varus and valgus stress tests are performed, during which the arm is loaded as shown in Figure 1. The assessment of joint instability is based on the perception of the examining physician. This manual procedure leads to a high degree of subjectivity in the diagnosis and requires significant experience and expertise.

Accurate assessment of joint instability remains a clinical challenge when conventional static imaging fails to represent pathological motion under physiological loading. This discrepancy is particularly evident in joints whose biomechanics depend on complex three‐dimensional interactions and ligamentous constraints. Videofluoroscopy is often used to clinically asses instabilities but it suffers from overprojection and a lack of quantitative data.^8^

Four‐dimensional computed tomography (4D-CT) has emerged as a powerful modality to visualize joint kinematics in real time, capturing motion rather than reconstructing it from sequential static poses.^8^ The clinical relevance of this technology has been most convincingly demonstrated at the distal radioulnar joint (DRUJ), where instability often manifests exclusively under load.^9^

Several groups have established 4D-CT as a tool for DRUJ analysis. This was supported by the development of CT-compatible motion and stress devices that transformed DRUJ assessment from subjective interpretation toward quantitative 3D biomechanics, laying the conceptual foundation for load-controlled, CT-based functional imaging.^10-12^

In contrast, 4D-CT at the elbow, though increasingly recognized as valuable for dynamic elbow disorders has remained limited to active patient motion without standardized loading.^13-15^ Clinical series have demonstrated that 4D-CT meaningfully alters diagnosis and management in cases of mechanical impingement, post-traumatic block, and suspected residual instability. In a case series of patients with suspected dynamic elbow pathology, Seah et al. showed that 4D-CT led to a change in diagnosis in 57% of cases and to an adjustment in therapeutic approach in over 50% of cases, with improved visualization of the pathological anatomy achieved in almost all cases.^14^

However, current approaches rely on voluntary motion that cannot be quantified and do not reproduce the stress conditions that are fundamental to clinical elbow examination. Varus-valgus stress testing, pivot-shift-type maneuvers, and the evaluation of subtle posterolateral rotatory instability (PLRI) depend inherently on external forces whose magnitude, direction, and time course are neither standardized nor measurable within existing imaging protocols.

On the elbow there was a recently published work from Lund et al. about valgus stress MRI to detect insufficiencies of the medial collateral ligament (MCL). The group showed that with getting the patient into a so called FEVER-position with flexed elbow and an external rotated shoulder there were more MCL injuries detected than in a normal positioned MRI.^16^ This shows that diagnosing is more precise with a targeted stress test and an adequate imaging.

The primary objective of the present work is to address this gap. We describe the design, fabrication, and initial evaluation of a CT-compatible, force-controlled motion device for standardized stress testing of the elbow during 4D-CT acquisition. We defined the acronym MELBO (Moved Elbow Laxity Biometric Optical) diagnostic tool as the project name. The task of the device is to enable controlled movement of the forearm in three degrees of freedom and to measure the resulting forces on the elbow relevant to diagnosis during this movement. In combination with innovative 4D-CT, the device provides a novel perspective in the joint kinematics under load. Before being used in a clinical setting, the device is intended to contribute to a deeper understanding of elbow joint function and its behavior in the case of instability through cadaver studies. The potential of this approach provides strong motivation for further investigations aimed at advancing both the diagnosis and treatment of elbow instabilities.

Forster et al developed an initial functional prototype, which serves as the foundation for this work.^17^ In the present paper, the follow-up prototype with adjusted kinematics and improved stability is introduced, the validation of the force sensor is addressed, and the first cadaver test results are presented.

## The Issue of Metal in CT

**Figure 2.**
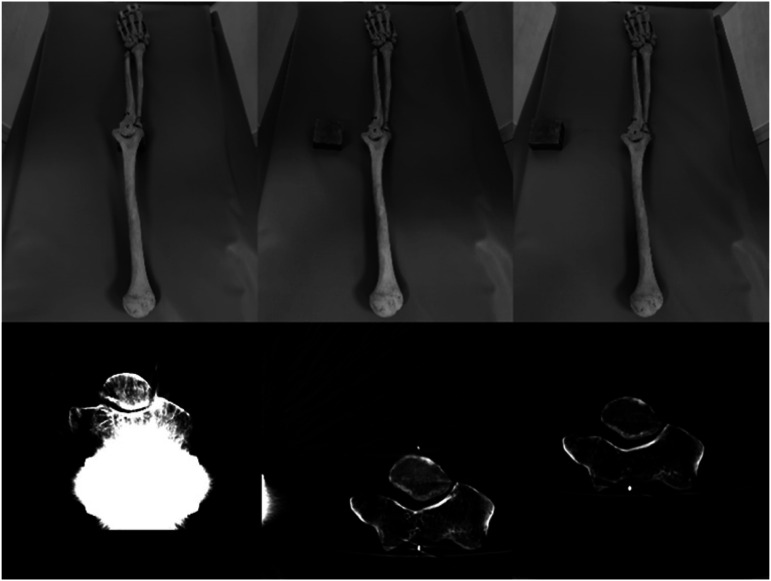
Test to assess the influence of the distance between the elbow joint and metallic objects on the generation of artifacts. A steel block was placed directly beneath the elbow joint, followed by two scans with increasing distances. The interference potential decreases significantly with greater distance

Computed tomography (CT) is an indispensable radiation-based imaging technique in clinical practice. X-rays are emitted by a radiation source, partially absorbed by the object under examination, and detected by a sensor positioned behind it. Since different tissues (such as muscle, cartilage, and bone) have varying absorption characteristics, they can be distinguished from one another. Metals, by contrast, exhibit absorption capacities that are orders of magnitude higher.^18^ Consequently, the design requirement for the apparatus was to be free of metal components. The first prototype was manufactured almost entirely from plastic using 3D printing.^17^ However, this low absorption capacity comes at the cost of significant disadvantages regarding stability, precision, and reliability compared to solutions using metal components. To enable a well-founded assessment of the interference potential of metal in the Siemens NAEOTOM Alpha, a test was conducted in Figure 2. The goal was to determine how close metal could be placed to the elbow joint without rendering the static X-ray image unusable. These findings were crucial for the subsequent design phase of the device. It can be concluded that the potential for interference decreases surprisingly rapidly as the distance increases. Therefore, in this design, metal was used but placed with a distance as large as possible from the elbow joint. By contrast, all components near the joint were designed to be metal-free.

## Design

**Figure 3.**
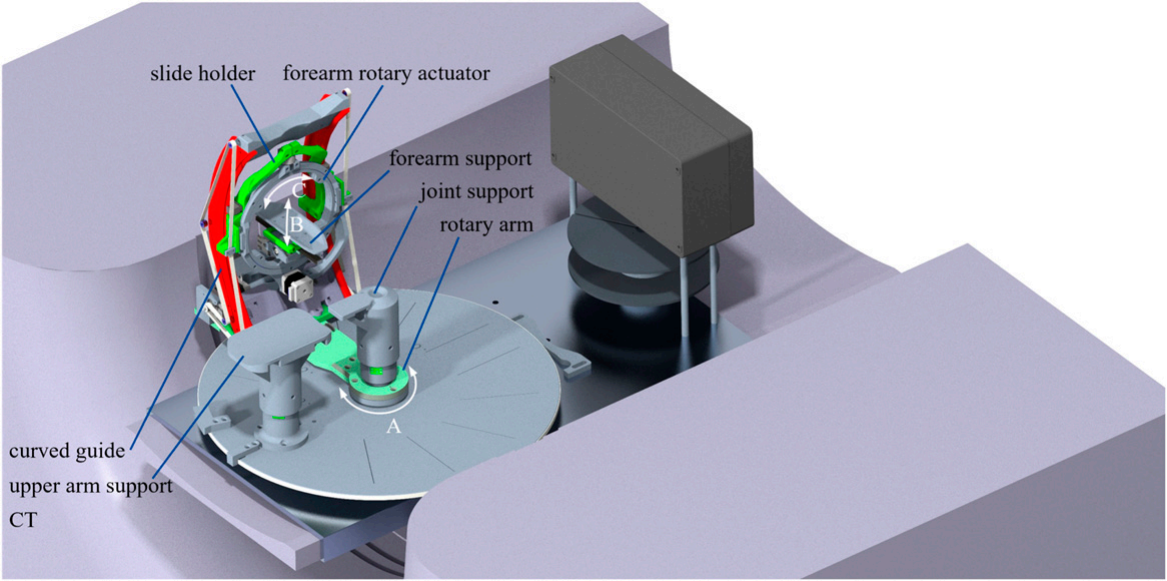
CAD model of the elbow treatment device in 4D-CT. The 4D-CT is displayed horizontally sectioned, showing only the lower half. The three degrees of freedom (A) (flexion/extension), (B) (varus-valgus stress), and (C) (pronation/supination) are indicated

In Figure 3, the setup is shown in detail. A photo of a cadaver arm placed in the device can be seen in Figure 8. The upper arm is aligned along the longitudinal axis of the 4D-CT and secured on the upper arm support, ensuring that the elbow joint is positioned as centrally as possible on the joint support. The forearm is guided through the forearm rotary actuator and placed on the forearm support, where it is fixed in place. The device is designed to move the forearm in three degrees of freedom during the scan. These are flexion/extension (motion A in Figure 3), varus-valgus stress (motion B in Figure 3), and pronation/supination (motion C in Figure 3). All motion axes are driven by stepper motors via timing belts. The force required for varus-valgus stress is crucial for diagnosis and will be measured using a force sensor. A comprehensive description of the device’s requirements was provided by Forster et al.^17^ The key components are presented in detail below.

### Flexion/Extension

When considering the right arm placed in the device (like in Figure 8), the flexion/extension is a circular motion of the hand in the horizontal plane around the elbow joint as the center (motion A in Figure 3). Motion A results from the rotation of the device’s rotary arm. The rotary arm is secured at the center of the circular base plate by a non-metallic ball bearing and can rotate around its vertically aligned axis.

### Varus-Valgus Stress

**Figure 4.**
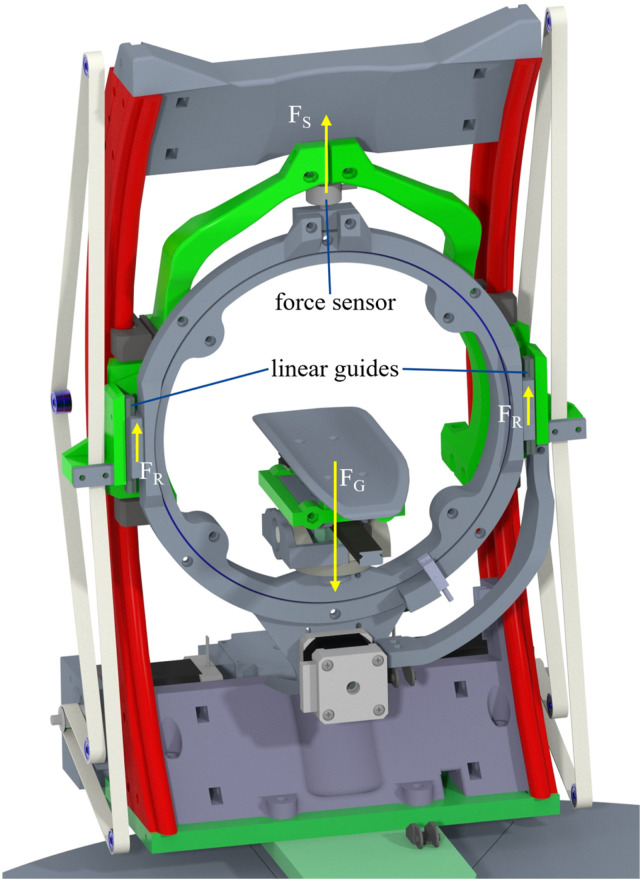
The integration of the force sensor can be seen. The force required for varus-valgus stress can flow exclusively through the force sensor using linear guides. Only the horizontally tangential force for flexion/extension ((A) in Figure 3) is transmitted through the linear guides. F_G_ = gravitational force/varus-valgus force, F_R_ = friction force, F_S_ = sensor force

The varus-valgus stress involves a circular motion of the hand in a vertical plane around the elbow joint as the center (motion B in Figure 3). Varus-valgus stress is realized by raising or lowering the forearm rotary actuator via two timing belts. This motion is guided using a curved guide. The curved guide ensures that the forearm support tilts during the lifting movement. This means that the line of action of the force sensor is always perpendicular to the forearm. This simplifies the calculation of the torque around the elbow joint, as the angle between the force and the lever (arm) is always 90°. In addition, the distance between the point of force application and the pivot point, the elbow joint, remains constant regardless of the lifting position. With a purely vertical lifting guide, this distance would increase from the neutral position. The forearm rotary actuator is coupled to the slide holder only through the force sensor and two linear guides, so that the entire force applied for the varus-valgus stress flows through the sensor. In Figure 4, the integration of the force sensor in combination with the ball screw linear guides is visible.

### Pronation/Supination

The pronation/supination of the forearm, when considering an arm placed in the device, is a rotation of the forearm around its longitudinal axis (motion C in Figure 3). This results from the rotation of the forearm rotary actuator, which is mounted for rotation by a thin-ring bearing. Upon closer inspection of the forearm rotary actuator in Figure 4, a circular gap in the assembly can be seen, which marks the transition from the inner to the outer ring of the bearing. The inner ring, which is firmly connected to the forearm support, is thus rotatably mounted.

### Force Sensor

The current medical standard is to perform the varus-valgus stress tests manually (Figure 1) and to base the assessment of instability of the elbow joint on the force exerted at a specific arm position. Applied to the arm placed in the device, the forearm is raised or lowered at specific flexion/extension angles through the varus-valgus stress motion, thus being loaded in the same way as shown in Figure 1. The force applied during this process is to be measured. A well-thought-out integration of the force sensor is essential for reliable measurement values. The total force required for the varus-valgus stress must flow through the force sensor. Furthermore, the force sensor should only be loaded in its measuring direction (tension or compression), meaning it should not experience shear, bending, or torsion. A design fulfilling these conditions is shown in Figure 4. Since the linear guides are aligned exactly parallel to the force sensor’s line of action, the vertical force to be measured for varus-valgus stress can only flow through the force sensor.

### Forearm Support

**Figure 5.**
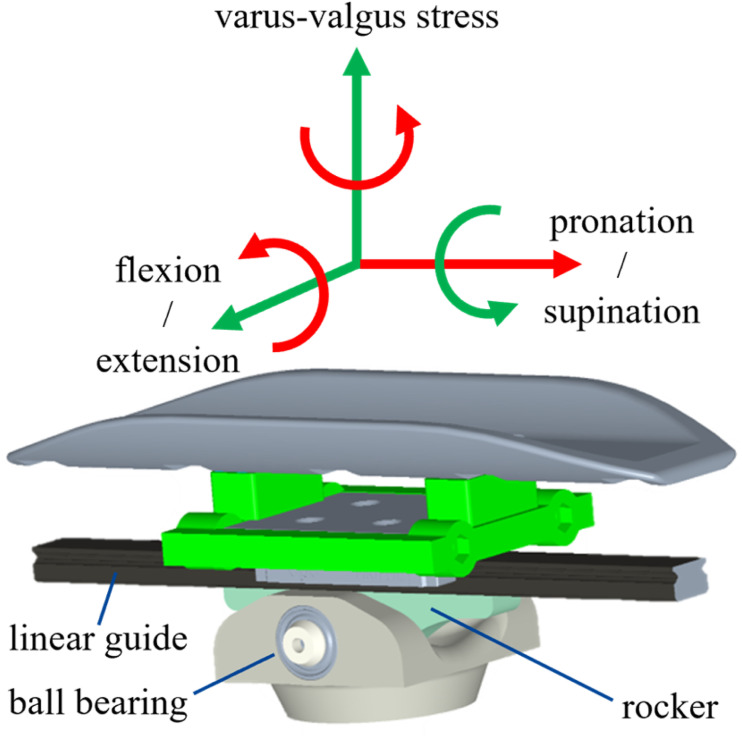
The forearm support only transmits the forces and torques necessary for the movement. When considering a right arm placed on the forearm support, needed force/torque transmission degrees of freedom of the forearm are shown as green arrows, and released ones red regarding to the arm movement. The structural design prevents the transmission of undesirable forces and torques

To move the arm, the corresponding forces and torques must act on it. These are transmitted at the contact point to the device, the forearm support (Figure 5). It is also important not to load the arm in an undesirable way. The basic idea of the assembly is to transmit all the necessary forces and torques to the arm while releasing all undesired forces and torques, preventing them from being transmitted. A body in three-dimensional space has 6 degrees of freedom: three translational and three rotational. However, the arm is only to be moved in 3 degrees of freedom: flexion/extension, varus-valgus stress, and pronation/supination (motion A, B, and C in Figure 3). The required forces and torques (two forces, one torque) for these movements are marked in green in Figure 5. This leaves two torques and one force that should not be transmitted from the forearm support to the arm. These are marked in red. In particular, an axial force should be prevented, as this would cause pain for patients later. However, bending torques should also not act on the forearm. Therefore, it is logical to release these degrees of freedom through corresponding elements to prevent such force or torque transmission from the device to the arm. In Figure 5, each released degree of freedom is assigned a corresponding construction element. The ball bearing rocker prevents torque transmission around the flexion/extension axis. The axis of the rocker is precisely parallel to the flexion/extension axis. The rocker is, in turn, rotatably mounted around the varus-valgus stress axis. The ball bearing is located beneath the rocker and is not visible in Figure 5. This prevents any bending load on the arm around the vertical varus-valgus stress axis. The most crucial consideration is preventing an axial load on the forearm. For this purpose, the forearm support is placed on a ball screw linear guide, which is aligned towards the elbow joint. Thus, force transmission along the pronation/supination axis is excluded. This ensures optimal force and torque transmission for the movement of the arm. Undesired loads are structurally excluded.

### Upper Arm Fixation

As previously explained, the diagnostic process involves performing varus-valgus stress tests. These are used to assess the instability of the elbow joint complex, during which the forearm is moved accordingly (Figure 1). Since the focus is on detecting joint instability, the upper arm must be fixed to prevent it from following the forearm movement. Only under this condition can any observed forearm motion be attributed exclusively to the instability of the elbow joint.

**Figure 6.**
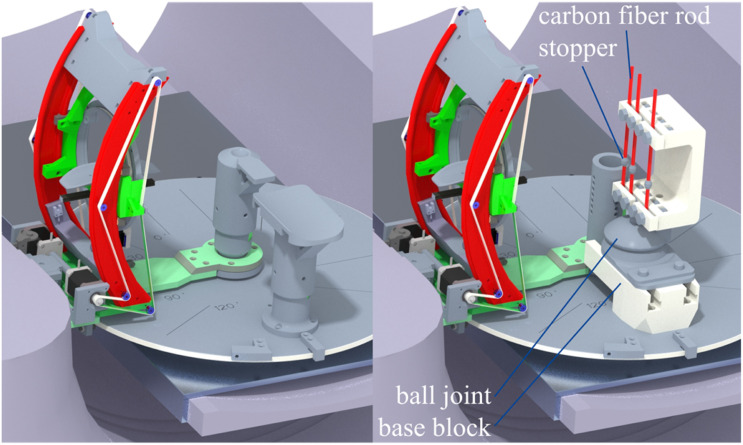
Upper arm fixation for patient studies (left) and cadaver studies (right)

Two different fixation systems for the upper arm were developed, one for later patient studies and another for cadaver studies. Figure 6 shows these.

The upper arm support on the left in Figure 6 was developed for patient studies. It consists of a height-adjustable shell in which the upper arm is placed and secured using two Velcro straps. In addition, the patient’s shoulder joint and bodyweight provide further stabilization of the upper arm and prevent axial movement of the upper arm or rotation around its longitudinal axis in the tissue. However, it must be examined whether this type of fastening is sufficient and to what extent suboptimal fastening affects the results.

This stabilizing function is absent in cadaver studies. Consequently, during initial tests, the humerus rotated along its longitudinal axis within the surrounding muscle tissue during specific forearm movements, rendering the measurements unusable. This issue is addressed by the upper arm fixation shown on the right in Figure 6. In cadaver studies, the humerus is vertically drilled in two locations, carbon rods are inserted through the boreholes and clamped into the dedicated fixation. Axial displacement of the bone along the rods is prevented by stoppers glued to the rods. Since the accuracy of the borehole positioning is inherently limited, the substructure allows as many degrees of freedom for fine adjustment as possible. The fixation can be tilted in all directions via a ball joint. On the base block, it can also be shifted radially and rotated around the vertical axis. This ensures optimal placement of the elbow joint at the center of rotation of the device, even if the boreholes are not ideally positioned.

### Control Unit

The control unit contains all the device’s electronics, including power supplies, microcontroller (Raspberry Pi), stepper motor drivers, force sensor evaluation, and an analog-to-digital converter (ADC) for the sensor signal. In addition, a Wi-Fi router is mounted on the control unit, enabling wireless control of the device via a mobile device. This is important because no one is allowed to be in the immediate vicinity of the 4D-CT during the scan. For this purpose, the Raspberry Pi provides a graphical user interface via NodeRed, which can be accessed from any device on the same network via a browser. This allows the arm to be moved manually over the network interface. It is also possible to start CT-synchronous predefined movement sequences. This causes the device to start moving at the same time as the 4D-CT scan. To do this, a light sensor is attached to a warning light on the 4D-CT and monitored by the Raspberry Pi. When the scan starts, the status of the warning light changes and the device begins to move. The force measurements can then be assigned to the corresponding points in time of the 4D-CT scan.

## Validation of the Device

### Force Measurement

To ensure the integrated force sensor’s proper functioning, it is calibrated using several approximately equally heavy metal blocks (m ≈ 300 g) as reference weights, which are successively placed on the forearm support. The sensor outputs a pre-filtered voltage signal directly proportional to the applied force. The microcontroller reads this voltage via an ADC. The proportionality constant must be determined for the fully assembled device. This constant indicates the change in voltage per unit of force and is required to convert the measured voltage signal into a force value.

**Figure 7.**
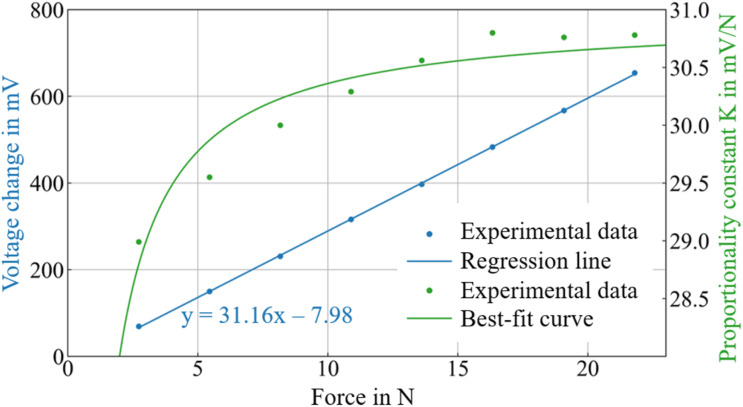
Voltage change (blue) and proportionality constant (green) of the force sensor as a function of the force applied to the forearm support. The slight deviation of the green measurement points from the best fit curve is acceptable. Of interest is the position of the horizontal asymptote, as this corresponds to the ideal proportionality constant. Values from Table 1

First, the sensor’s no-load voltage is recorded. Then, the weights are stacked one by one onto the forearm support, and the corresponding voltage change values are measured. If the sensor functions correctly, plotting the measured voltages against the applied forces yields a straight line through the origin, with its slope corresponding to the proportionality constant K. The evaluation is shown in Figure 7 in blue.

Figure 7 shows the experimentally determined data pairs in blue. A regression line is plotted. The data points lie approximately on a straight line. However, a notable feature is the y-axis intercept, which lies significantly in the negative range.

Table 1 lists the measurement values. The force value $F_{G}$ corresponds to the sum of all weights placed on the forearm support multiplied by the gravitational acceleration g. The voltage value ΔU corresponds to the measured voltage minus the force sensor’s no-load voltage.

**Table 1.** Force Sensor Evaluation

Test weight	1	1-2	1-3	1-4	1-5	1-6	1-7	1-8
F_G_ in N	2.73	5.45	8.17	10.89	13.61	16.33	19.08	21.80
ΔU in mV	79	161	245	330	416	503	587	671
K_calculated_ in mV/N	28.99	29.55	30.00	30.29	30.56	30.80	30.76	30.78
K_regression_ in mV/N	31.16							
ΔK in %	-6.98	-5.16	-3.73	-2,79	-1.94	-1.17	-1.29	-1.24

Table 1 shows that the calculated proportionality constant K increases with greater loading of the force sensor and appears to approach a limiting value. The reason for this behavior is the frictional force acting within the ball bearing linear guides (Figure 4). The relevant forces are illustrated. This frictional force, *F*_*R*_*,* consistently counteracts the gravitational force of the weights by a fixed amount. It originates from the preload of the linear guides, which results in an essentially constant frictional resistance.

As a result, the calculated proportionality constant is always based on a weight force $F_{G}$ that is offset by this friction, since part of the gravitational force is dissipated via the linear guides and does not reach the force sensor. Table 1 allows the assumption that the ratio of the frictional force $F_{R}$ to the gravitational force $F_{G}$ is crucial, as the error decreases with increasing force $F_{G}$ . The following section derives this relationship.

(1) $$K_{calculated\;}=\frac{\Delta U}{\;F_{G}}$$

Although $F_{G}$ is used in (1) for the calculation, only the portion reduced by the frictional force $F_{R}$ , namely $F_{S}$ , acts on the force sensor (Figure 4). The following applies:

(2) $$F_{G}=F_{S}+F_{R}$$

(3) $$K_{calculated}=\frac{\;\Delta U}{F_{S}+F_{R}}$$

The gravitational force $F_{G}$ used in the calculation is thus divided into a portion that actually acts on the sensor and a portion absorbed by the ball screw linear guides. Rearranging (3) results in

(4) $$K_{calculated}=\frac{\Delta U}{F_{S}}\;\frac{\;1}{1+\frac{F_{R}}{F_{S}}}=\frac{\Delta U}{F_{S}}\;\frac{F_{S}}{F_{S}+F_{R}}$$

If $F_{S}$ in the second term of (4) is replaced by (2), (5) results:

(5) $$K_{calculated}=\frac{\Delta U}{F_{S}}\;\frac{F_{G}-F_{R}}{F_{G}}=\frac{\Delta U}{F_{S}}\;\left(1-\frac{F_{R}}{F_{G}}\right)$$

The term $\frac{\Delta U}{F_{S}}$ corresponds to the ideal proportionality factor $K_{ideal}$ , which results from dividing the corresponding voltage value by the friction-corrected, actual force $F_{S}$ acting on the sensor. This value is of particular interest.

(6) $$K_{calculated}=K_{ideal}\;\left(1-\frac{F_{R}}{F_{G}}\right)$$

Now, we have an expression that illustrates the impact of the frictional force on the constant $K_{ideal}$ to be determined. As the gravitational force $F_{G}$ increases, the influence of the constant frictional force $F_{R}$ on the calculation decreases, and the calculated value approaches the ideal value $K_{ideal}$ . This fact is illustrated in Figure 7 in green. The value pairs of the experimentally determined proportionality factor $K_{calculated}$ are shown as a function of the corresponding gravitational force $F_{G}$ . Equation (6) is also shown in green. The parameters $K_{ideal}$ and $F_{R}$ are chosen such that the curve best fits the green experimental points (Python curve_fit-function from SciPy-Library^19^). The found factor $K_{ideal}$ is 30.95 mV/N, which is very close to the slope of the blue regression line in Figure 7 (31.16 mV/N). Also noteworthy is the found value for $F_{R}$ of 0.19 N. With this consideration, the friction in the ball screw linear guides can now be approximated, which helps evaluate the quality of the force measurement.

### First Cadaver Test

**Figure 8.**
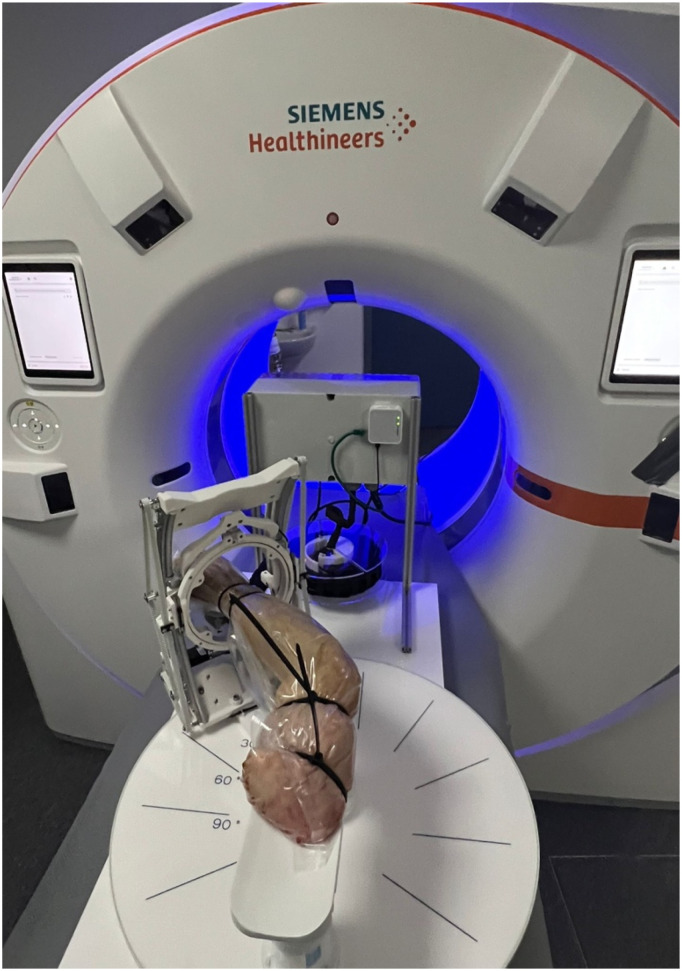
An arm inserted into the device during the first test. Here, the upper arm was secured with cable ties, which proved to be insufficient. The in Figure 6 presented holder is therefore used for cadaver arms

Finally, an initial cadaver test was conducted to test the practical applicability of the prototype (Figure 8). For testing the right arm of a 78-year-old male without any reported elbow disorders like osteoarthritis, previous fractures or dislocations was used.

Due to its compact size, the elbow joint fits entirely within the axial 6 cm imaging coverage of the Siemens NAEOTOM Alpha used in this study. Therefore, unlike larger anatomical structures, the joint does not need to be moved axially through the imaging area during scanning. Instead, it is positioned centrally within the field of view and moved by the developed device during the scan. This results in a 3D recording of the moving elbow joint. For Scan acquisition, images were obtained at 90 kV with an effective tube current-time of 20 mAs, a rotation time of 0,25 seconds, using a Br44 reconstruction kernel and reconstructed with a slice thickness of 0,8mm. Dynamic CT data were reconstructed at a temporal sampling rate of approximately 2,6 volumes per second based on a cycle time of 0,38 seconds.

**Figure 9.**
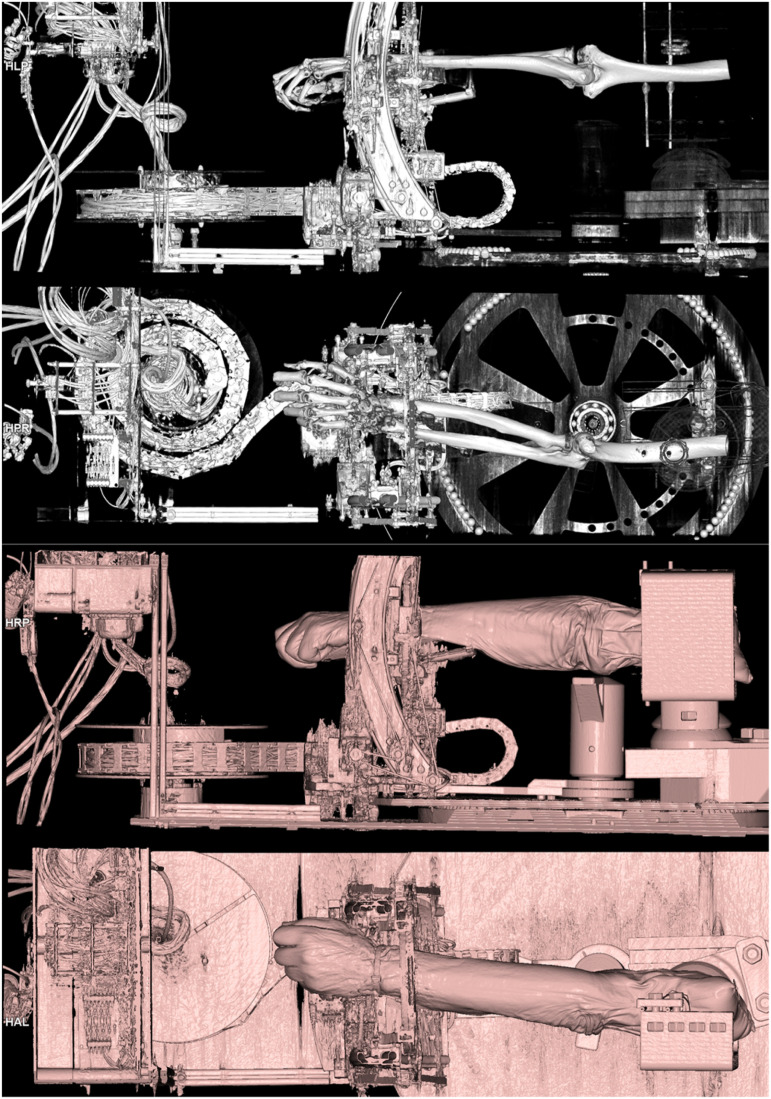
Volume rendered CT-images in top and side view of the entire device during the first cadaver test, reconstructed once based on the bone and once on the skin. The correct positioning of the arm in the device can be verified this way. In the top view perspective, a slightly suboptimal joint positioning at the joint support can be observed

First the physicians positioned the arm in the device. The entire apparatus was moved through the imaging field during a scan to verify correct alignment. The reconstructed images are shown in Figure 9. Achieving ideal arm positioning presents a significant challenge. The goal is to align the arm so that the kinematics of the device precisely match those of the arm. For this, the joint must be located exactly at the center of rotation of the device. This process requires skill and typically several iterations.

**Figure 10.**
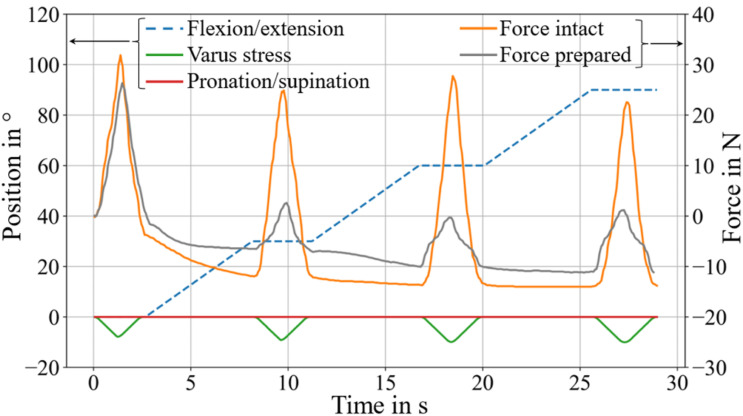
Comparison of varus stress forces of the same arm, first in the intact state and then after ligament transection, using an identical testing sequence consisting of varus stress tests at flexion/extension angles of 0°, 30°, 60°, and 90° in a neutral pronation/supination position. A decrease in force can be observed in the prepared, unstable joint, which was to be expected. The variation in force between the varus stress tests during extension suggests a slightly suboptimal positioning of the arm in the device

Figure 10 shows the analysis of measurement data of the first cadaver test. The varus force on the arm was measured once in its intact state (orange) and once after preparation with severed ligaments (grey).

Sectioning of the lateral collateral ligament complex (LCLC) was performed after testing the intact state via a standard Kocher approach. Following blunt dissection through the Kocher interval along muscle fiber orientation, the LCLC was completely detached from its humeral insertion.

Varus stress was applied at flexion/extension angles of 0°, 30°, 60°, and 90° (blue). The pronation/supination angle remained constant at 0° (red). Each sequence starts at a flexion/extension position of 0°. The varus stress (green) begins moving downward, causing the opposing resistance force to increase accordingly. At the maximum varus stress deflection of −10°, the motion reverses and returns to the initial position. Then, a new flexion/extension angle is set, and the procedure is repeated. Comparing the force curves of the intact and the prepared, unstable elbow joint reveals a lower resistance force for the unstable joint at identical device positions. This is consistent with the expected behavior in the presence of joint instability and thus confirms the device’s basic functionality.

A noticeable change in force is observed during the flexion/extension movements between the varus stress tests, especially during movement from 0 ° to 30 ° flexion angle (Figure 10). This could indicate suboptimal positioning of the arm in the device and must be further investigated in future medical studies. In initial cadaver studies, placement was considered good if the joint apparatus did not move out of the image area during flexion. However, the quantitative impact of misplacement on the measured force values must be investigated in greater depth. Avoiding these is difficult, but algorithms that factor out misplacement are thinkable and planned.

**Figure 11.**
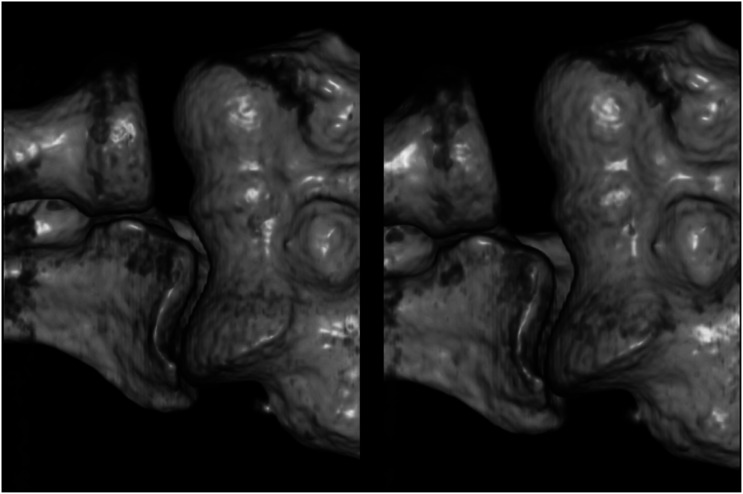
4D-CT imaging of the same arm as in Figure 10, intact (left) and prepared with severed ligaments (right), under varus stress. A clear difference in joint space width is visible, confirming the presence of instability

Besides the forces, a significant change in the elbow joint space can be observed during the varus stress test in the 3D video of the 4D-CT scan (Figure 11). Now, meaningful sectional planes can be defined within the 3D model, based on which these changes in the joint space can be quantitatively evaluated.

The measured force, device positional data, and 4D-CT images showing the corresponding joint space can now be evaluated in combination to identify patterns that may enable diagnostic conclusions. An optimal method for assessing the data to achieve diagnostic significance must be developed in detail.

## Discussion

The aim of this study was to describe the development, assembly and initial testing of a fully functional second prototype of the MELBO diagnostic tool for cadaveric investigations building upon the initial prototype.^17^ The original requirement of complete metal-free construction was relaxed to allow the use of metallic components for functional elements. Initial cadaver testing confirmed improved mechanical robustness, allowed acquisition of 4D-CT datasets suitable for qualitative and quantitative assessment of elbow joint stability and enabled simultaneous recording of force measurements. No relevant imaging artifacts attributable to the diagnostic tool were observed. The device is compact and fits entirely into the gantry of the CT-Scanner.

Particular emphasis was placed on validation of the force measurement system, including assessment of internal friction within the device. The measured frictional forces were approximately 0.2 N, indicating a high level of measurement precision and substantially exceeding the reproducibility expected from manual force application by a physician.^20^

Previous studies have explored the use of diagnostic devices for objective assessment of joint instability. Yoshioka et al. demonstrated that a TELOS device can apply different loads to the elbow joint at varying degrees of elbow flexion. Using ultrasound-based measurement of joint space width, they identified significant differences between groups.^21^ However, the TELOS device allows loading of the joint only in one position at a time. In contrast, the MELBO diagnostic tool enables flexible modification of movement and stress application according to the clinically suspected injured elbow ligament.

In combination with a 4D-CT this allows three-dimensional visualization of joint motion and may facilitate detection of subtle instability patterns.

Podgorski et al. assessed elbow laxity and varus-valgus range of motion using the UB-01 measuring device. The device consists of a seated testing station with an adjustable boom and by a two-piece foam-lined clamp at the distal arm and an adjustable belt positioned in the axilla, while the system continuously records angular deviation of the forearm and the corresponding applied torque.^22^ Due to its design, this device does not appear suitable for use within a 4D-CT environment.

A key advantage of the MELBO diagnostic tool is its ability to center the elbow joint within the scanning range while moving the joint at a defined speed, thereby allowing CT acquisition protocols to be matched to the motion characteristics of the device. This is relevant in the context of previous elbow 4D-CT studies using freehand patient-performed motion. Seah et al reported images loss and motion artifacts when patients performed elbow maneuvers freely after prior instruction, particularly when movements were performed too quickly or too slowly. They further described that instructing patients in advance and achieving an optimal movement speed was time consuming.^13,14^ In contrast, device guided motion with the MELBO may reduce the dependence on patient training, standardize movement during acquisition and improve reproducibility in future clinical studies.

Schnetzke et al. evaluated elbow laxity in a cadaveric model with sequential soft-tissue injury and demonstrated that dynamic fluoroscopy can discriminate between different grades of collateral ligament compromise.^23^ However, their experimental setup allowed stress application only in discrete static positions and does not appear to be transferable to in vivo assessment or compatible with continuous motion acquisition as required for four-dimensional CT.

Following cadaveric testing, clinical studies using our MELBO diagnostic tool will be required to determine whether it enables improved diagnostic assessment of elbow instability. Harmonization of scan protocols with the velocity and motion characteristics of the system will be addressed in future investigations, including dedicated evaluation of image quality and radiation exposure in clinical 4D-CT protocols. Further investigations will be necessary to identify clinically relevant motion patterns and to optimize acquisition protocols accordingly.

## Future Perspectives

Safety mechanisms are currently being developed and integrated into the device to support future patient use. These include a force-controlled varus-valgus stress test that allows the definition of the maximum permitted effective force. In addition, the use of EMG sensors is currently being investigated to detect patient protective muscle reflexes in response to pain and stop or reverse the movement when required.

The next step will be to gain further practical experience with the device in additional cadaver studies, critically evaluate the measured data, and derive new insights from these investigations.

The combined analysis of 4D-CT images, force measurement data, and positional data from the device is expected to provide substantial diagnostic potential. A method must be developed to evaluate the measurement data quantitatively, contributing to diagnosis.

In future studies involving larger cadaveric or clinical cohorts, quantitative analysis of 4D-CT datasets in combination with synchronized force and positional measurements will become increasingly relevant. Automated or AI-based approaches may represent a useful methodological extension for pattern recognition and objective assessment of dynamic elbow instability once sufficiently robust training datasets are available. By enabling reproducible, quantifiable loading conditions in real time, the device could provide a foundation for objective biomechanical evaluation of elbow instability, facilitate future normative and pathological comparisons, ultimately support evidence-based clinical decision-making and might prevent unnecessary surgery in the future.

## Conclusion

The present study demonstrates the technical feasibility of device-guided simultaneous force and positional measurement with acquisition of diagnostically usable image data without relevant device-related artifacts. Quantitative correlation of imaging-derived instability parameters with applied load and elbow position remains the subject of future investigations with further cadaveric validation and subsequent clinical evaluation to determine its potential role in objective, reproducible assessment of elbow instability.
